# Economic Burden of Human Immunodeficiency Virus and Hypertension Care Among MOPHADHIV Trial Participants: Patient Costs and Determinants of Out-of-Pocket Expenditure in South Africa

**DOI:** 10.3390/ijerph22101488

**Published:** 2025-09-25

**Authors:** Danleen James Hongoro, Andre Pascal Kengne, Nasheeta Peer, Kim Nguyen, Kirsty Bobrow, Olufunke A. Alaba

**Affiliations:** 1 Health Economics Unit, School of Public Health, Faculty of Health Sciences, University of Cape Town, Anzio Road, Observatory, Cape Town 7925, South Africa; olufunke.alaba@uct.ac.za; 2 Non-Communicable Diseases Research Unit, South African Medical Research Council, Cape Town 7925, South Africa; 3 Department of Medicine, Faculty of Health Sciences, University of Cape Town, Cape Town 7925, South Africa; 4 African Population and Health Research Center, Kitisuru, Manga Close, Kirawa Road, Nairobi 00100, Kenya

**Keywords:** HIV, hypertension, multimorbidity, economic burden, out-of-pocket costs, South Africa, health equity, integrated care, catastrophic health expenditure, MOPHADHIV trial

## Abstract

**Background:** Human immunodeficiency virus and hypertension increasingly co-occur in South Africa. Despite publicly funded care, patients with multimorbidity face high out-of-pocket costs, yet limited evidence exists from the patient perspective. **Purpose:** To quantify the economic burden of comorbid HIV and hypertension, assess predictors of monthly out-of-pocket costs, and explore coping mechanisms. **Methods:** We conducted a cross-sectional analysis using patient-level data from the Mobile Phone Text Messages to Improve Hypertension Medication Adherence in Adults with HIV (MOPHADHIV trial) [Trial number: PACTR201811878799717], a randomized controlled trial evaluating short messages services adherence support for hypertension care in people with HIV. We calculated the monthly direct non-medical, indirect, and coping costs from a patient perspective, valuing indirect costs using both actual income and minimum wage assumptions. Generalized linear models with a gamma distribution and log link were used to identify cost determinants. Catastrophic expenditure thresholds (10–40% of monthly income) were assessed. **Results:** Among 683 participants, mean monthly total costs were ZAR 105.81 (USD 5.72) using actual income and ZAR 182.3 (USD 9.9) when valuing indirect costs by minimum wage. These time-related productivity losses constituted the largest share of overall expenses. Regression models revealed a strong income gradient: participants in the richest quintile incurred ZAR 131.9 (95% CI: 63.6–200.1) more per month than the poorest. However, this gradient diminished or reversed under standardized wage assumptions, suggesting a heavier proportional burden on middle-income groups. Other socio-demographic factors (gender, employment, education) not significantly associated with total costs, likely reflecting the broad reach of South Africa’s primary health system. Nearly half of the participants also reported resorting to coping mechanisms such as borrowing or asset sales. **Conclusions:** Comorbid HIV and hypertension impose substantial patient costs, predominantly indirect. Income disparities drive variation, raising equity concerns. Strengthening integrated human immunodeficiency virus—non-communicable diseases care and targeting financial support are key to advancing South Africa’s Universal Health Coverage reforms.

## 1. Introduction

Co-prevalent human immunodeficiency virus (HIV) and hypertension present a growing challenge for health systems in sub-Saharan Africa (SSA), where services have historically focused on communicable diseases, particularly HIV. In South Africa the epicentre of the global HIV epidemic and home to the largest antiretroviral therapy (ART) programme, declines in HIV-related mortality have given rise to a rapidly aging cohort of people living with HIV (PLWH). This population is increasingly affected by non-communicable diseases (NCDs), particularly hypertension [1,2]. This dual burden complicates clinical management and places additional stress on already fragmented and under-resourced health systems [3].

South Africa’s health system is based on universal access to primary care, with chronic conditions like HIV and hypertension largely managed through publicly funded services [4]. However, despite this coverage, patients still face substantial out-of-pocket (OOP) costs for non-medical expenses such as transport, food, and productivity losses, reflecting gaps in financial protection [5]. Such expenditures can be impoverishing, especially for low-income households, where even small recurrent costs may exceed catastrophic thresholds (10–40% of monthly income) [6,7]. International and South African evidence highlights that OOP spending reflects both barriers to access and inequities in health systems performance [8]. OOP spending, therefore, serves as a proxy for both access barriers and inequities in health system performance. Within this framework, we conceptualize OOP costs as indicators of financial risk and equity, aligning with the goals of South Africa’s Universal Health Coverage (UHC) reforms [9].

Existing literature highlights the substantial costs faced by patients managing chronic conditions in low-resource settings, with determinants including income level, distance to healthcare facilities, and comorbidity complexity [10,11]. Across SSA, OOP expenditures for HIV care, though partially offset by free ART remain catastrophic for 10–25% of households [6,7]. These figures vary by context; South Africa’s universal primary care and access to WHO-recommended essential medicine coverage likely mitigates some costs relative to countries such as Malawi or Uganda, where patients often pay out of pocket for a broader range of services [9,12]. For hypertension, OOP costs are less documented but often include frequent clinic visits and unsubsidized medications, disproportionately affecting low-income populations [13,14]. Catastrophic spending is exacerbated by multimorbidity, yet integrated HIV/NCD care models show promise in reducing costs. Still, patient-level data on the combined economic burden of HIV and hypertension remain scarce, particularly in South Africa’s UHC reform context.

While medical consultations, diagnostics, and medicines are generally provided at no cost in the public sector [4,15], significant indirect and non-medical expenses persist. Patients continue to face significant indirect and non-medical costs, particularly for transport, food, and lost wages [5,8]. Even for free healthcare services such as ART, total OOP costs can consume 15–30% of a household’s monthly income [5]. When HIV and hypertension coexist, this cumulative burden is amplified, yet few studies have quantified its full impact [1,5].

Evidence from South Africa and comparable settings reinforces this gap. Studies in KwaZulu-Natal highlight transport and lost income as the dominant cost drivers for PLWH [5]. In West and Central Africa, high levels of catastrophic health expenditure have been reported despite free provision of ART: nearly 60% of PLHIV in Lagos, Nigeria incurred catastrophic costs [6], while in Cameroon, 20% of outpatients and 67% of inpatients faced catastrophic expenditure, with transport and borrowing emerging as key determinants [7]. These findings demonstrate that subsidization of HIV services alone is insufficient to prevent financial hardship and underscore the importance of evaluating patient-incurred costs in South Africa. Methodologically, much of this literature has relied on descriptive analysis, with limited application of econometric approaches suited to skewed, non-negative cost distributions. Generalized linear models (GLMs) with gamma distribution and log link are increasingly recognized as more robust, yet remain underutilized in SSA cost studies [16].

Against this backdrop, this study contributes by analysing baseline data from the Mobile Phone Text Messages to Improve Hypertension Medication Adherence in Adults with HIV (MOPHADHIV) trial, a cluster-randomized evaluation of integrated HIV and hypertension care in South Africa. Specifically, we aimed to (i) quantify direct non-medical, indirect, and coping costs; (ii) identify socio-demographic predictors of elevated monthly OOP expenditure; and (iii) assess coping strategies such as borrowing, asset sales, or skipping treatment. These findings aim to inform UHC policy reforms and improve financial protection for patients managing multimorbidity in resource-limited settings.

## 2. Materials and Methods

### 2.1. Study Design and Analytical Framework

This study analysed patient-incurred costs using baseline data from the MOPHADHIV trial, a randomized controlled trial evaluating the effect of short message services (SMS) adherence support for hypertension care in PLWH across public primary health facilities in the Western Cape Province of South Africa. The trial was conducted between 2021 and 2024 and registered on Pan-African Clinical Trial Register, trial number: PACTR201811878799717 [17].

The MOPHADHIV trial employed a cluster-randomized design across four community health centres in the Cape Town metropolitan area. While this design ensured a robust evaluation framework, it also meant that our findings are most representative of urban settings with relatively well-resourced primary healthcare services, which may not fully capture the experiences of patients in rural or under-served provinces [4]. Of the 697 eligible participants approached, 683 (98%) completed the baseline cost survey, reflecting a low non-response rate. However, income data were only available for 436 participants, which may limit the precision of socioeconomic stratification. We addressed this by conducting available-case analysis but acknowledge that the missingness may not be random. In addition, all cost, time, and coping variables were self-reported, which introduces the possibility of recall error and social desirability bias, as noted in similar patient cost studies in South Africa [5].

Data were collected from four selected community health centres: Mitchells Plain Community Day Centre (CDC), District 6 Community Health Centre (CHC), Mfuleni CHC, and Kraaifontein CHC. We adopted a cross-sectional study design, restricted to participants with complete cost and socioeconomic data. Our primary objective was to quantify the economic burden of care and identify the determinants of monthly OOP expenditure, as well as to assess coping strategies and cost burden thresholds. Analyses were conducted in *Stata version 17* (StataCorp LLC, College Station, TX, USA) and followed a three-stage framework: (i) cost construction, (ii) regression estimation, and (iii) financial burden assessment.

### 2.2. Costing Perspective and Definitions

The study adopted a patient perspective to estimate the financial burden of care, focusing on three categories of patient-incurred costs: direct non-medical costs, indirect costs, and coping costs (see Supplementary File S1 for survey items and measurement details). All cost, time, and coping variables were self-reported with a one-month recall period, consistent with standard patient cost surveys in South Africa. This monthly recall frame reduces recall error relative to longer periods but may still introduce reporting bias, which we acknowledge as a limitation. Travel and clinic waiting times were similarly self-reported for the most recent visit and assumed to occur once per month, consistent with routine chronic care schedules. While this reflects typical patient experience, we note that time estimates were not externally validated (e.g., GPS tracking or time logs), which may introduce measurement error. All costs were adjusted for inflation using year-specific deflators (2021 = 1.21; 2022 = 1.14; 2023 = 1.09; 2024 = 1.00), converted to 2024 South African Rand (ZAR), and subsequently to United States Dollars (USD) using a fixed exchange rate of ZAR 18.50/USD.

Cost data were highly right-skewed, a common feature of patient-incurred cost datasets [16]. To account for this, we applied log-transformation of cost variables as a standard normalization technique in health economics. However, our primary regression analyses employed GLMs with a gamma distribution and log link, which directly accommodate skewed, non-negative cost outcomes without requiring transformation of the dependent variable [17,18,19]. The log-transformed variables were retained only for sensitivity analyses using ordinary least squares (OLS) regression.

We acknowledge that more flexible approaches, such as generalized additive models (GAM) and generalized additive models for location, scale, and shape (GAMLSS), may offer additional advantages by capturing non-linearity and complex distributional features directly, without modifying the original cost data [20]. While these approaches were beyond the scope of this study, they represent a methodological limitation of our analysis and a potential direction for future research.

Direct medical costs including consultation fees, diagnostics, and medications were not included in this analysis. This exclusion reflects the South African health system context, where public primary care services are free at the point of use, particularly for chronic conditions such as HIV and hypertension [4,5]. Therefore, most participants incurred no formal charges for medical services, and our focus on direct non-medical, indirect, and coping costs better captures the financial burden faced by patients.

Cost variables were computed using the following formulas:

1. ***Direct Non-Medical Costs***

(1) $$C_{dnmed}=C_{food}+(2*\;C_{travel\;})$$

2. ***Indirect Costs***

- *Actual wage (self-reported):*

(2) $$C_{indirect\_actual}=\left(\;\frac{T_{travel}+T_{clinic}}{60}\right)*\left(\frac{I_{monthly}}{176}\right)$$

- *Minimum wage (ZAR 28.79/hr):*

(3) $$C_{indirect\_minimum\_wage}=\left(\frac{T_{travel}+T_{clinic}}{60}\right)*28.79\;$$

where $T_{travel}$ and $T_{clinic}$ are in minutes; $I_{monthly}$ is monthly income in ZAR.

3. ***Coping Costs***

Coping costs refer to financial strategies patients use to manage healthcare-related expenses when regular income or savings are insufficient. These include the monetary value of loans taken, savings withdrawn, assets sold (e.g., household items), and any additional costs incurred from modifying dietary habits due to illness. These were captured through self-reported responses to structured questions (see Section H of Supplementary File S1).

(4) $$C_{coping\_actual}=C_{borrowed}+C_{interest}+C_{withdrawal}+C_{assets}+C_{diet}$$

4. ***Total Monthly Cost***

(5) $$C_{total_{actual}}=C_{dnmed}+C_{indireect_{actual}}+C_{coping}$$

(6) $$C_{total\_minimum\_wage}=C_{dnmed}+C_{indirect\_minimum\_wage}+C_{coping}$$

All models were estimated on costs adjusted to 2024 ZAR and USD, as described in Section 2.2. For regression analyses, cost variables were log-transformed to correct for right-skewed distributions:

(7) $$\ln(C_{total}+1)\;$$

### 2.3. Covariates and Socioeconomic Measures

Demographic and socioeconomic variables were constructed and recoded as follows:

∘ Gender: Binary (0 = Male, 1 = Female)
∘ Age group: 24–34, 35–49, 50–54, 55+ years
∘ Employment: Full-time, part-time/self-employed, unemployed/unable to work
∘ Education: No schooling, primary (Grade 1–7), secondary (Grade 8–12)
∘ Household size: Small (≤3), medium (4–6), large (≥7)
∘ Travel time (roundtrip): <30 min, 30–60 min, >60 min

Socioeconomic status (SES) was derived from self-reported income. Using *Stata*’s *xtile* command, we categorized participants into five income quintiles (poorest to richest). This approach enabled stratification of the cohort into distinct SES classes for analysis. Income data were available for 436 of 683 participants (64%). Non-response was primarily due to participants choosing not to disclose income. Socioeconomic status quintiles were therefore derived using available-case analysis without imputation. Because income non-response may not be random, SES-related results should be interpreted with caution.

### 2.4. Model Construction and Specification

The determinants of total patient-incurred costs were estimated using GLMs with a gamma distribution and log link function, selected for their appropriateness in modelling right-skewed, non-negative cost data. Model construction followed a systematic approach: (1) Variable selection was guided by theoretical relevance (e.g., income, employment) and prior literature on healthcare expenditure in low-resource settings; (2) Distributional assumptions were validated through graphical inspection (e.g., Q-Q plots) and comparison of Akaike Information Criterion (AIC)/Bayesian Information Criterion (BIC) values across alternative families (gamma vs. inverse Gaussian); (3) Link function suitability was confirmed via Pearson residuals analysis, with the log link ensuring predicted costs remained positive; (4) Covariate coding adhered to interpretable referent categories (e.g., poorest income quintile as baseline). For robustness, we compared GLM results with OLS on log-transformed costs, with both specifications including identical covariates. Model fit was assessed using scaled deviance statistics and residual plots, with no evidence of overdispersion.

#### Econometric Specification

We estimated the determinants of total patient-incurred costs GLMs with a gam-ma distribution and log link, which are well-suited for modelling right-skewed, non-negative data. For robustness, we also estimated OLS regressions on log-transformed costs. Average marginal effects (AMEs) were computed to facilitate interpretation. To evaluate financial protection, we assessed cost burden as a share of monthly income and calculated catastrophic health expenditure at thresholds of 10%, 20%, 25%, 30%, and 40%. The full econometric specifications, including GLM, OLS, and catastrophic expenditure equations, are provided in Supplementary File S2.

## 3. Results

### 3.1. Descriptive Statistics

The study initially included 697 participants. Most participants were middle-aged adults (35–49 years; 52.7%, 95% CI: 48.9–56.4) or in the pre-retirement (50–54 years; 20.4%, 95% CI: 17.5–23.5) and senior (≥55 years; 20.5%, 95% CI: 17.7–23.7) age groups (Table 1). Gender distribution was skewed, with 80.6% (95% CI: 77.5–83.4) identifying as female. Regarding employment, 62.1% (95% CI: 58.4–65.7) were unemployed or unable to work, while 25.5% (95% CI: 22.3–28.9) reported full-time employment.

Among the 588 participants with education data, 77.6% (95% CI: 74.4–80.6) had attained secondary education, 18.2% (95% CI: 15.5–21.3) had only primary education, and 4.2% (95% CI: 2.9–5.9) had no formal schooling (Table 1). Household sizes were mostly small (1–3 members; 46.1%, 95% CI: 42.4–49.8) or medium (4–6 members; 44.3%, 95% CI: 40.7–48.1), while 9.6% (95% CI: 7.6–12.0) lived in large households (7+ members), possibly reflecting urban housing patterns. Income data, available for 436 participants, indicated a relatively even distribution across quintiles, with slightly higher representation in the poorest (Q1: 20.2%, 95% CI: 16.7–24.2) and second (Q2: 23.4%, 95% CI: 19.7–27.6) groups. The richest quintile (Q5) comprised 19.0% (95% CI: 15.6–23.0).

### 3.2. Socioeconomic Gradients in Cost Composition

A socioeconomic gradient is evident in the composition of patient-incurred costs (Table 2). Among the poorest quintile (Q1), coping costs made up the largest share (61.4%), indicating heavy reliance on strategies such as borrowing or dietary compromise. In contrast, for the richest quintile (Q5), productivity-related indirect costs dominated (72.3%), reflecting greater time losses due to travel and clinic visits. Direct non-medical costs (e.g., transport, food) remained relatively consistent but modest across all quintiles.

**Figure 1:**
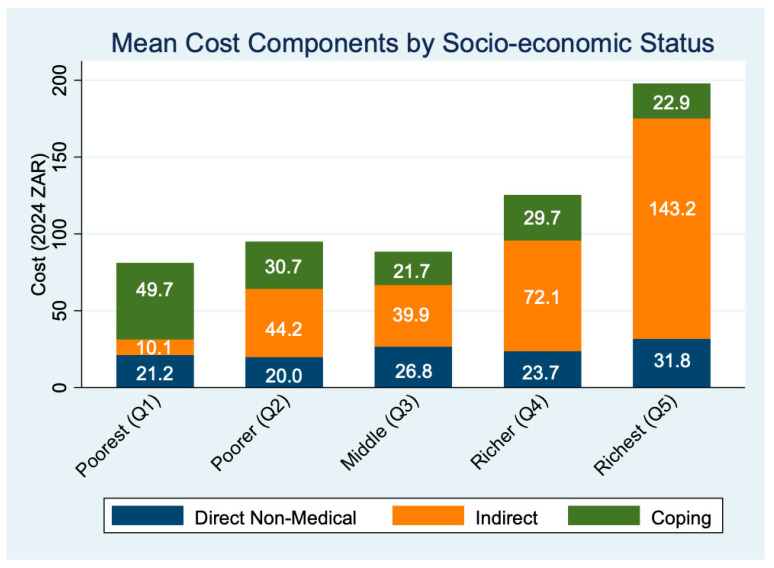
Mean costs by socio-economic status.

Figure 1 shows that mean total costs increased with SES, from ZAR 81.07 in Q1 to ZAR 197.93 in Q5, primarily due to rising indirect costs (from ZAR 21.19 in Q1 to ZAR 175.01) in Q5. On average, participants reported 47.6 min of travel time and 2.3 h of waiting per clinic visit, underscoring the time burden of accessing care.

**Figure 2:**
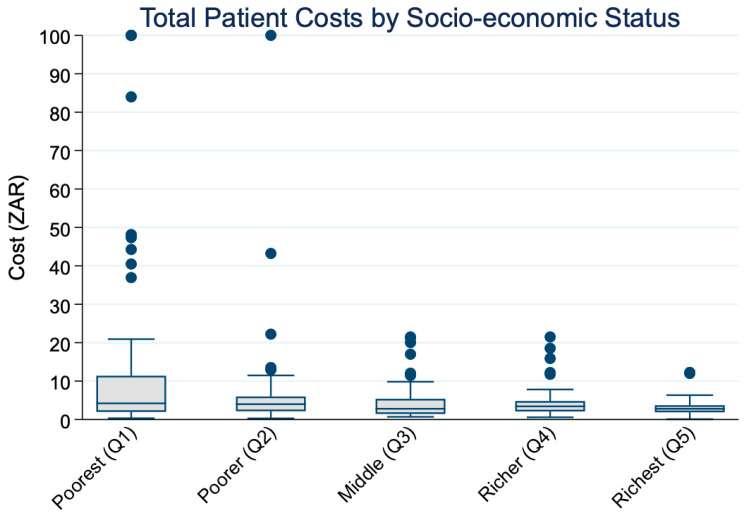
Distribution of median total patient costs across socio-economic quintiles.

Figure 2 displays the distribution of total patient costs by socio-economic status. A clear downward trend is visible, with higher median costs among the Q1 and progressively lower costs among richer quintiles. This visual pattern is supported by Cuzick’s non-parametric test. As shown in Table 3, Cuzick’s non-parametric test for trend confirmed a significant decreasing trend in median total costs across income quintiles (z = −4.0, SE = 8.2, *p* < 0.001). The mean response score was highest in the poorest group (14.3 ZAR) and declined to 3.0 ZAR in the richest quintile, suggesting an inverse relationship between socio-economic status and out-of-pocket patient costs. These results appear inconsistent with Figure 1 and Table 2, which show rising mean costs across income quintiles. The difference reflects the skewed distribution of cost data: wealthier individuals incur very high costs that inflate the mean, while the median, less sensitive to outliers, declines across quintiles. Taken together, these findings highlight that central tendency measures lead to different interpretations; mean costs suggest greater absolute expenditures among the rich, while medians indicate a relatively heavier burden among the poor.

Direct non-medical costs, primarily transportation expenses, had a mean of ZAR 26.3 (USD 1.4) per month, although the median was substantially lower at ZAR 0.8 (USD 0.0), indicating that half of the participants incurred minimal out-of-pocket transport costs (Table 4). The distribution was highly skewed, with the 90th percentile at ZAR 68.4 (USD 3.7) and a maximum of ZAR 436.0 (USD 23.6), suggesting that a subset of patients bore disproportionately high transport-related costs.

Indirect costs, reflecting productivity losses, varied significantly by valuation method. When based on self-reported income losses, the mean was ZAR 47.7 (USD 2.6) and the median was ZAR 28.5 (USD 1.5). However, when valued using the minimum wage to estimate opportunity costs, the mean increased to ZAR 124.2 (USD 6.7), with a median of ZAR 108.9 (USD 5.9). This substantial increase highlights the considerable time burden of care-seeking, even for those not formally employed. The 75th percentile under this valuation reached ZAR 162.1 (USD 8.8), indicating that at least one-quarter of participants experienced productivity losses equivalent to more than half a day’s wages per month. Coping costs had a median of zero, indicating no reported financial coping strategies for at least half of participants. Nonetheless, the mean was ZAR 31.7 (USD 1.7), with maximum costs reaching ZAR 1635.0 (USD 88.4), reflecting that a minority of individuals resorted to potentially harmful financial measures to manage care costs (Table 4).

Total monthly costs averaged ZAR 105.8 (USD 5.7) under the actual cost assumption and ZAR 182.3 (USD 9.9) under the minimum wage valuation for indirect costs. The 72% difference between these estimates underscores the importance of valuation method in assessing the true economic burden of chronic disease management. The distribution of total costs was also right-skewed, with the 90th percentile at ZAR 233.0 (USD 12.6) and ZAR 313.3 (USD 16.9) for actual and minimum wage-based valuations, respectively. Maximum costs exceeded ZAR 1600.0 (USD 89.0), illustrating that a small proportion of patients experienced potentially catastrophic health expenditures (Table 4).

### 3.3. Regression

Table 5 reports the AMEs from GLMs with a gamma distribution and log link, estimating the association between socio-demographic characteristics and monthly patient-incurred costs. Across both models, most socio-demographic variables including gender, employment status, education level, household size, age group, and travel time were not statistically significantly associated with patient costs. For instance, compared to males, female participants incurred ZAR 4.9 less (95% CI: −42.5 to 32.7) in Model 1 and ZAR 6.8 less (95% CI: −44.5 to 30.9) in Model 2; both differences were statistically insignificant. Similarly, being self-employed or unemployed, relative to full-time employment, was not significantly associated with monthly costs in either model. Education levels and household size also showed no consistent associations, although participants from large households (7+ members) incurred ZAR 257.0 less in Model 2 (95% CI: −512.2 to −0.0), a marginally significant result.

A clear income-related gradient was observed. In Model 1 (actual income valuation), participants in the fourth quintile incurred ZAR 49.6 (95% CI: 11.5–87.7) more per month than those in Q1, while those in Q5 incurred ZAR 131.9 (95% CI: 63.6–200.1) more. In Model 2 (minimum wage valuation), this pattern was attenuated or reversed. Specifically, participants in Q3 incurred significantly lower costs than the poorest (ZAR −47.3; 95% CI: −92.3 to −2.2), while estimates for richer quintile (Q4) and Q5 were not statistically significant.

Travel time greater than 60 min was associated with ZAR 42.0 higher costs in Model 2 (95% CI: −8.9 to 92.9), though not statistically significant. As indicated in Table 5, statistical significance was observed only for income quintiles. In the minimum wage–based model, participants in Q3 had significantly lower costs compared to the Q1. In contrast, in the actual income model, participants in Q4 and Q5 incurred significantly higher costs than Q1. No other socio-demographic variables were statistically significant. This pattern aligns with South Africa’s coverage scheme, where medical costs are covered by the state, leaving income-related productivity losses as the primary driver of differences in patient-incurred expenditures.

Table 6 presents the AMEs of socio-demographic factors on OOP healthcare expenditure, estimated from GLMs with a gamma distribution and log link. Overall, few variables were statistically significant predictors of OOP costs. Gender was not associated with significant differences; female participants incurred ZAR 4.6 less costs per month than males (95% CI: −45.2 to 36.1). Employment status, education level, household size, and age group similarly showed no statistically meaningful associations. For example, individuals with secondary education incurred ZAR 2.3 more costs than those with no schooling (95% CI: −89.3 to 93.8), and participants from large households (7+ members) had a non-significant increase in ZAR 7.3 (95% CI: −61.0 to 75.6).

In contrast, there was clear evidence of an income-related gradient in monthly OOP costs. Compared to individuals in the Q1, those in the Q4 incurred significantly higher costs (AME: ZAR 50.8, 95% CI: 12.9 to 88.7, *p* = 0.009), while participants in the Q5 had the largest incremental cost (AME: ZAR 128.2, 95% CI: 63.0 to 193.4, *p* < 0.001). This pattern may partly reflect greater choice autonomy among higher-income individuals, for example, the ability to opt for additional services, purchase preferred food items, or choose faster (but costlier) transport options. These findings demonstrate that income is the most consistent socio-demographic correlate of OOP healthcare expenditure in this sample.

### 3.4. Sensitivity Analysis

**Figure 3:**
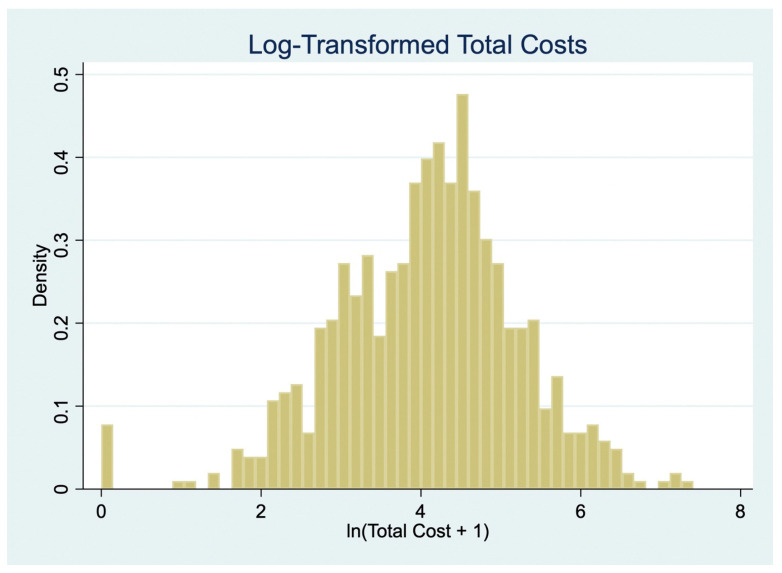
Log-transformed total costs.

Figure 3 displays the distribution of log-transformed total patient costs (ln [Total Cost + 1]), which approximates a normal distribution and supports the use of linear regression for sensitivity analysis. Table 7 presents the results from two OLS models regressing log-transformed total costs on socio-demographic variables. Model 1 uses actual reported indirect costs, while Model 2 substitutes these with minimum wage-based imputed costs.

In Model 1, higher household income was positively associated with higher log-transformed total costs. For example, compared to individuals in the Q1, those in poorer quintile (Q2), Q3, Q4, and Q5 had progressively higher coefficients ranging from β = 0.8 to β = 1.7 (all *p* < 0.001). Since these models use a log-transformed dependent variable, coefficients are interpreted as semi-elasticities: a coefficient of 0.8 corresponds to approximately a 122% increase in total costs (exp(0.8)–1), while a coefficient of 1.7 corresponds to an approximate 447% increase. These findings are consistent with the GLM marginal effects analysis, highlighting a socio-economic gradient in patient costs when actual income is used to value time. Model 1 explained 28% of the variation in log-transformed costs (R^2^ = 0.28).

In contrast, when indirect costs were estimated using the minimum wage approach (Model 2), the income gradient was attenuated or reversed. For instance, individuals in the Q3 had significantly lower costs compared to the Q1 (β = −0.3, 95% CI: −0.5 to −0.0, *p* < 0.05), which corresponds to a 26% decrease in costs (exp(−0.3)–1). This suggests that when opportunity costs are standardized, lower- and middle-income individuals may appear to bear a proportionally greater burden. The explanatory power of Model 2 was substantially lower (R^2^ = 0.06), indicating that minimum wage-based estimates may obscure socio-economic variation.

Other covariates were largely not significantly associated with total costs in either model. Exceptions include household size and travel time in Model 2. Participants from large households (7+ members) incurred significantly lower costs compared to those from small households (β = −0.3, 95% CI: −0.5 to −0.0, *p* < 0.05; ≈26% decrease), while those who travelled more than 60 min to access care reported higher costs (β = 0.3, 95% CI: 0.0 to 0.5, *p* < 0.05; ≈35% increase). Gender, employment status, education level, and age group were not significantly associated with costs in either specification.

It is important to note that these OLS models were included only as sensitivity analyses. Our primary results are based on GLMs with a gamma distribution and log link, which directly model skewed cost data without transformation. The apparent contradiction between Cuzick’s trend test (Table 3) and the regression models arises from differences in statistical focus: Cuzick’s test evaluates medians, which declined with income, while GLM and OLS estimate mean effects, which increased with income due to high outliers in wealthier groups. Taken together, these approaches provide complementary insights, highlighting higher absolute expenditures among the rich but proportionally greater burdens on the poor.

## 4. Discussion

This study aimed to quantify the economic burden of accessing and receiving care for hypertension among PLWH in South Africa, assess predictors of monthly OOP expenditure, and explore coping mechanisms used by patients. By adopting a patient-level perspective and leveraging detailed cost data from the MOPHADHIV trial, our findings offer new empirical insights into the financial challenges faced by individuals managing multiple chronic conditions in a resource-constrained setting. Despite both hypertension and HIV services being offered free at the point of care, our findings show that PLWH with comorbid hypertension still incur non-trivial non-medical, indirect, and coping-related costs. When productivity losses are valued using the minimum wage, average monthly expenditures remain meaningful, underscoring the ongoing economic burden of managing chronic multimorbidity even within a publicly funded health system. Although these costs were not catastrophic for most participants, they underscore the importance of protecting lower-income patients from cumulative financial strain. These figures are consistent with findings from Uganda and Kenya, where PLWH with multimorbidity similarly reported monthly cost burdens that exceeded 10% of household income, particularly when transport, lost income, and supplementary medicine costs were included [21,22].

Our disaggregated data reveal that indirect costs (primarily time lost to care-seeking) are the single largest cost component, echoing prior studies showing that health-related time loss is a critical but often underestimated driver of patient economic burden [2,23]. Notably, even among unemployed individuals, opportunity costs remain high, underscoring the hidden productivity burden associated with routine clinic visits [24]. Our regression models show a clear socioeconomic gradient in patient-incurred costs: wealthier patients incurred significantly higher absolute expenditures, particularly when using actual reported income to calculate productivity losses. For example, individuals in the Q5 spent more per month than those in the Q1, a statistically significant difference that persisted across multiple model specifications. These findings are consistent with national-level data indicating that hypertension care in South Africa results in higher total costs among more affluent patients, likely due to increased use of diagnostics and medications [25]. This may also reflect greater system navigation ability and financial flexibility among higher-income individuals, consistent with fundamental cause theory and evidence linking higher SES to greater health literacy and service use [26].

Our findings highlight the unequal distribution of financial burden in the care of PLWH with comorbid hypertension, where wealthier patients incur higher absolute costs, yet lower-income households experience greater relative strain. While Cuzick’s trend test suggested declining central costs with income, regression models captured the influence of extreme expenditures among wealthier groups. These results underscore the dual nature of financial burden: higher spending among the rich, but heavier proportional impact on the poor. Sensitivity analyses using minimum wage valuations shifted the apparent burden toward middle-income groups. This may reflect persistent SES-related differences in health-seeking behaviour, but also highlights the influence of valuation methods on how relative burden is interpreted. When adjusting for wage differentials, the burden appears to shift disproportionately toward lower-income patients particularly those in the middle-income quintiles who may not qualify for social protection but still face barriers to care [1,24]. These findings underscore the importance of considering both absolute and relative cost burdens in contexts of high-income inequality and informal labour markets.

Although the median coping cost was zero, nearly half of participants reported some form of coping expenditure. Notably, 40 individuals (9.6%) experienced catastrophic health spending exceeding 10% of their income, with a smaller subset of 11–12 individuals (2.6–2.9%) reporting costs above 30% to 40% of their monthly income. These outliers show that a meaningful proportion of patients still face financial hardship, even in a system with free public sector care. While our findings indicate that a smaller proportion of patients reported borrowing or selling assets to cover healthcare costs, such coping strategies were still present and may carry longer-term risks to household financial stability. In comparison, studies from settings with less comprehensive public health coverage such as Zimbabwe have reported substantially higher rates, with over 25% of patients resorting to asset sales or borrowing to afford chronic disease care [27]. These mechanisms may have lasting repercussions on household financial security and elevate vulnerability to future health shocks [5].

The presence of extreme cost outliers even among the poorest SES groups reinforces the idea that catastrophic expenditure is not limited to higher earners. It can also reflect structural and geographic barriers such as poor transport infrastructure, fragmented healthcare service delivery, and the lack of integrated care [24,28]. Surprisingly, most socio-demographic predictors including gender, education, and employment were not significantly associated with patient costs. This aligns with prior work in Kenya and South Africa, which found that SES, rather than demographic variables per se, was the most consistent predictor of financial burden [4,15,22]. However, travel time was marginally significant in sensitivity models, supporting the role of geographic access as a structural determinant [1,29].

These findings support the need for integrated healthcare models that combine HIV and NCD care to reduce patient time and transport costs. Although integration initiatives exist in South Africa, they are often inconsistently implemented and limited in scope, highlighting the need for more systematic and patient-centred approaches [2,28]. In the MOPHADHIV trial, HIV and hypertension services were co-located within primary care facilities. However, integration remained partial, characterized by separate registers, clinical teams, and follow-up systems. This siloed structure contributed to duplicate visits and fragmented care, inflating both direct and indirect patient costs. Strengthening integration could reduce cumulative time losses, streamline service delivery, and improve multimorbidity management without increasing patient burden [23]. The observed income gradient in patient-incurred costs suggests that lower-income individuals may face proportionally higher financial strain, even if absolute costs remain modest. These findings highlight the potential value of exploring targeted financial protection strategies such as transport support or differentiated subsidies to enhance equity within the health system. In addition, incorporating indirect costs into benefit design and economic evaluations may help ensure that reforms under South Africa’s UHC agenda are responsive to the time and productivity losses experienced by patients managing multiple chronic conditions.

### Strengths and Limitations

This study contributes a unique micro-costing dataset from a real-world primary care setting, using robust econometric methods including GLMs with gamma distribution and log link, and sensitivity checks using OLS models. Nevertheless, limitations include the reliance on self-reported income and time-use data, potential recall bias in coping cost reporting, and limited generalizability beyond public sector facilities in urban settings.

Potential biases may also arise from our choice of econometric models and from the data collection mechanism. While GLMs with a gamma distribution and log link are well-suited to skewed cost data, they assume variance proportional to the mean and may not fully capture heterogeneity in the sample. Our sensitivity analyses using OLS on log-transformed costs introduce different limitations, as coefficients reflect percentage changes rather than absolute differences. In addition, all cost, time, and coping variables were self-reported, which may be subject to recall errors and social desirability bias, consistent with other patient cost studies in South Africa [5]. These limitations should be considered when interpreting the findings.

Finally, while GLMs with gamma distribution and log link were our primary modelling strategy, supplemented by log-transformed OLS models, we acknowledge that these approaches rely on specific distributional assumptions. More flexible approaches, such as GAM and GAMLSS, have been shown to accommodate non-linearity and complex distributional features without requiring transformation [20]. Although beyond our current scope, these models may offer more nuanced insight into patient-incurred costs in future studies.

## 5. Conclusions

Free clinical services alone do not guarantee financial protection. In South Africa’s public sector, direct medical costs are covered by the state, yet patients continue to face a substantial financial burden through transport, food, and productivity losses. These non-medical and indirect expenditures undermine financial protection and highlight persistent inequities in a health system designed for universal coverage. Our findings demonstrate a clear socioeconomic gradient in out-of-pocket spending, with wealthier patients incurring higher absolute costs, but lower-income patients facing proportionally greater financial strain when opportunity costs are standardized. This underscores the need to address both absolute and relative measures of financial burden. Strengthening integrated platforms for PLWH with comorbid hypertension and reducing transport and opportunity costs are critical for advancing South Africa’s UHC reforms and ensuring equitable protection against the economic consequences of multimorbidity.

## Figures and Tables

**Table 1:** Descriptive Statistics of Study Participants (N = 697 unless otherwise specified).

Characteristic	N	%
Age Groups		
Young adults (24–34)	45	6.46
Middle-aged adults (35–49)	367	52.65
Pre-retirement (50–54)	142	20.37
Seniors (55+)	143	20.52
Gender		
Male	135	19.37
Female	562	80.63
Employment Status (N = 683)		
Employed full-time	174	25.48
Self-employed/part-time	85	12.45
Unemployed/unable to work	424	62.08
Education (N = 697)		
No schooling	29	4.16
Primary (Grade 1–7)	127	18.22
Secondary (Grade 8–12)	541	77.62
Household Size		
Small (1–3 members)	321	46.05
Medium (4–6 members)	309	44.33
Large (7+ members)	67	9.61
Income Quintile (N = 436)		
Poorest (Q1)	88	20.18
Poorer (Q2)	102	23.39
Middle (Q3)	81	18.58
Richer (Q4)	82	18.81
Richest (Q5)	83	19.04

**Table 2:** Percentage Contribution of Direct Non-Medical, Indirect, and Coping Costs to Total Patient-Incurred Costs by Socioeconomic Status (SES) Quintile.

SES Quintile	Direct Non-Medical (%)	Indirect (%)	Coping (%)
Poorest (Q1)	26.1	12.5	61.4
Poorer (Q2)	21.0	46.6	32.4
Middle (Q3)	30.3	45.2	24.5
Richer (Q4)	18.9	57.5	23.7
Richest (Q5)	16.1	72.3	11.6
Overall (All)	33.2	29.8	36.8

Note: Percentage composition of patient-incurred costs across SES quintiles, showing the relative share of direct non-medical costs (e.g., food and transport), indirect costs (e.g., productivity losses due to time), and coping costs (e.g., borrowing, asset sales, dietary compromise).

**Table 3:** Cuzick’s non-parametric test for trend in total patient costs across socio-economic quintiles (N = 419).

Socio-Economic Group	Number of Observations	Mean Response (Cost in ZAR)
Poorest (Q1)	72	14.33
Poorer (Q2)	102	5.85
Middle (Q3)	81	4.35
Richer (Q4)	82	4.10
Richest (Q5)	82	3.02

Note: Cuzick’s test for trend: z = −4.02, SE = 8.17, *p* < 0.001.

**Table 4:** Summary Statistics of Patient-Incurred Costs for Accessing and Receiving Care for hypertension by people living with HIV.

Cost Category	Mean (ZAR)	Std Dev (ZAR)	Median (ZAR)	P25 (ZAR)	P75 (ZAR)	P90 (ZAR)	Max (ZAR)	Mean (USD)	Std Dev (USD)	Median (USD)	P25 (USD)	P75 (USD)	P90 (USD)	Max (USD)
Direct Non-Medical Cost	26.29	46.08	0.82	0	37.06	68.4	436	1.42	2.49	0.04	0	2.00	3.70	23.57
Indirect Cost—Actual	47.71	57.39	28.49	16.6	55.58	106.94	577.67	2.58	3.10	1.54	0.90	3.00	5.78	31.23
Indirect Cost—Minimum Wage	124.23	79.58	108.86	67.99	162.14	217.05	548.12	6.72	4.30	5.88	3.68	8.76	11.73	29.63
Coping Cost	31.73	128.97	0	0	0	57	1635	1.72	6.97	0.00	0.00	0.00	3.08	88.38
Total Cost—Actual	105.81	150.34	62.86	27.02	117.18	232.96	1649.45	5.72	8.13	3.40	1.46	6.33	12.59	89.16
Total Cost—Minimum	182.34	160.33	144.97	93.62	214.44	313.29	1708.22	9.86	8.67	7.84	5.06	11.59	16.93	92.34

**Table 5:** Estimates of average marginal effects (AME) from generalized linear models with a gamma distribution and log link.

	Actual Cost [Model 1]			Minimum Wage [Model 2]		
Variable	AME ^1^	SE ^2^	95% CI ^3^	AME	SE	95% CI
Female (vs. Male)	−4.894	19.198	[−42.52, 32.73]	−6.794	19.216	[−44.46, 30.87]
Self-employed/part-time (vs. Full-time)	14.013	20.753	[−26.66, 54.69]	3.85	22.42	[−40.09, 47.79]
Unemployed/unable to work (vs. Full-time)	1.763	17.298	[−32.14, 35.67]	−7.958	21.123	[−49.36, 33.44]
Primary Education (vs. No Schooling)	−33.41	46.068	[−123.70, 56.88]	−37.359	49.23	[−133.85, 59.13]
Secondary Education (vs. No Schooling)	2.734	45.607	[−86.66, 92.12]	−11.028	48.427	[−105.94, 83.89]
HH Size: Medium (4–6) (vs. Small)	24.313	15.109	[−5.30, 53.93]	9.518	13.76	[−17.45, 36.49]
HH Size: Large (7+) (vs. Small)	9.538	35.807	[−60.64, 79.72]	−16.69	29.111	[−73.75, 40.37]
Middle-age (35–49) (vs. Young adults)	−22.762	39.415	[−100.01, 54.49]	−28.879	48.633	[−124.20, 66.44]
Pre-retirement (50–54) (vs. Young adults)	3.541	44.551	[−83.78, 90.86]	−12.662	50.958	[−112.54, 87.21]
Seniors (55+) (vs. Young adults)	4.877	42.492	[−78.41, 88.16]	−3.978	49.385	[−100.77, 92.82]
30–60 min (vs. <30 min)	−2.546	17.399	[−36.65, 31.56]	9.671	16.652	[−22.97, 42.31]
>60 min (vs. <30 min)	19.614	30.512	[−40.19, 79.42]	42.027	25.973	[−8.88, 92.93]
Income Q2 (vs. Poorest Q1)	12.015	20.263	[−27.70, 51.73]	−6.997	27.18	[−60.27, 46.28]
Income Q3 (vs. Poorest Q1)	14.365	17.697	[−20.32, 49.05]	−47.28	22.987	[−92.33, −2.23] *
Income Q4 (vs. Poorest Q1)	49.587	19.436	[11.49, 87.68] *	−25.88	24.072	[−73.06, 21.30]
Income Q5 (vs. Poorest Q1)	131.881	34.824	[63.63, 200.13] ***	−29.617	29.69	[−87.81, 28.57]

Model 1 uses actual indirect costs; Model 2 uses minimum wage-based indirect costs; *** *p* < 0.01, ** *p* < 0.05, * *p* < 0.1. ^1^ Average Marginal Effects. ^2^ Standard Error. ^3^ 95% Confidence Interval.

**Table 6:** Average Marginal Effects of Socio-Demographic Factors on Monthly Out-of-Pocket Expenditure (ZAR).

Variable	dy/dx	SE ^1^	z	*p*-Value	95% Confidence Interval	
Female (vs. Male)	−4.55	20.76	−0.22	0.827	−45.24	36.14
Self-employed/part-time (vs. Full-time)	15.54	21.56	0.72	0.471	−26.72	57.8
Unemployed/unable to work (vs. Full-time)	3.32	17.42	0.19	0.849	−30.82	37.46
Primary Education (vs. No schooling)	−34.95	46.97	−0.74	0.457	−127.01	57.12
Secondary Education (vs. No schooling)	2.27	46.71	0.05	0.961	−89.28	93.83
HH Size: Medium (4–6) (vs. Small)	23.38	15.13	1.54	0.122	−6.28	53.04
HH Size: Large (7+) (vs. Small)	7.28	34.86	0.21	0.835	−61.04	75.6
Middle-age Adults (35–49) (vs. Young adults)	−20.43	38.75	−0.53	0.598	−96.38	55.53
Pre-retirement (50–54) (vs. Young adults)	3.36	43.52	0.08	0.939	−81.94	88.66
Seniors (55+) (vs. Young adults)	1.67	41.81	0.04	0.968	−80.28	83.61
Income Q2 (vs. Poorest Q1)	14.16	21.75	0.65	0.515	−28.46	56.78
Income Q3 (vs. Poorest Q1)	16.99	17.78	0.96	0.339	−17.85	51.84
Income Q4 (vs. Poorest Q1)	50.78	19.33	2.63	0.009	12.89	88.67
Income Q5 (vs. Poorest Q1)	128.18	33.26	3.85	0.000	63	193.36

^1^ Standard Error.

**Table 7:** Determinants of Log-Transformed Patient Costs: Sensitivity Analysis Using Actual and Opportunity Cost Approaches.

Variable	Actual Cost Model (1)			Min Wage Cost Model (2)		
	Coefficient (β)	Robust SE	95% CI	Coefficient (β)	Robust SE	95% CI
Gender (ref: Male)						
Female	0.089	(0.165)	[−0.236, 0.413]	−0.011	(0.103)	[−0.213, 0.191]
Employment (ref: Full-time)						
Self-employed/part-time	0.079	(0.147)	[−0.210, 0.368]	0.016	(0.116)	[−0.212, 0.244]
Unemployed/unable to work	−0.161	(0.117)	[−0.391, 0.070]	−0.116	(0.105)	[−0.323, 0.091]
Education (ref: None)						
Primary (Grade 1–7)	0.248	(0.385)	[−0.510, 1.005]	0.026	(0.252)	[−0.469, 0.522]
Secondary (Grade 8–12)	0.398	(0.383)	[−0.356, 1.151]	0.095	(0.249)	[−0.394, 0.584]
Household size (ref: 1–3)						
Medium (4–6)	0.087	(0.102)	[−0.113, 0.287]	0.022	(0.068)	[−0.111, 0.156]
Large (7+)	−0.319	(0.193)	[−0.698, 0.060]	−0.257 *	(0.130)	[−0.512, −0.003]
Age group (ref: 24–34)						
Middle-age adults (35–49)	0.066	(0.251)	[−0.428, 0.560]	−0.019	(0.158)	[−0.330, 0.293]
Pre-retirement adults (50–54)	0.162	(0.281)	[−0.390, 0.714]	0.045	(0.174)	[−0.297, 0.387]
Seniors (55+)	0.266	(0.288)	[−0.299, 0.832]	0.185	(0.167)	[−0.144, 0.514]
Travel time (ref: <30 min)						
30–60 min	−0.022	(0.135)	[−0.288, 0.244]	0.094	(0.110)	[−0.123, 0.311]
>60 min	−0.001	(0.181)	[−0.357, 0.354]	0.276 *	(0.126)	[0.028, 0.524]
Income quintile (ref: Q1)						
Poorer (Q2)	0.839 ***	(0.204)	[0.437, 1.240]	0.012	(0.108)	[−0.201, 0.225]
Middle (Q3)	0.871 ***	(0.211)	[0.456, 1.285]	−0.256 *	(0.111)	[−0.474, −0.038]
Richer (Q4)	1.298 ***	(0.192)	[0.922, 1.675]	−0.058	(0.112)	[−0.278, 0.162]
Richest (Q5)	1.724 ***	(0.213)	[1.305, 2.143]	−0.128	(0.143)	[−0.408, 0.153]
Constant	2.793 ***	(0.544)	[1.724, 3.862]	4.863 ***	(0.338)	[4.199, 5.527]
Model statistics						
Observations	434			434		
R-squared	0.28			0.06		

Note: *** *p* < 0.01, ** *p* < 0.05, * *p* < 0.1.

## Data Availability

The data used and analyzed during the current study are not publicly available due to participant confidentiality and data protection agreements but may be made available from the corresponding author on reasonable request and with appropriate institutional approvals. Written informed consent was obtained from the patient(s), during the study.

## Supplementary Materials

The following supporting information can be downloaded at: https://www.mdpi.com/article/10.3390/ijerph22101488/s1, File S1: Patient cost survey; File S2: Econometric Specifications.
