# Correction: Hongoro et al. Economic Burden of Human Immunodeficiency Virus and Hypertension Care Among MOPHADHIV Trial Participants: Patient Costs and Determinants of Out-of-Pocket Expenditure in South Africa. *Int. J. Environ. Res. Public Health* 2025, *22*, 1488

**DOI:** 10.3390/ijerph23050631

**Published:** 2026-05-11

**Authors:** Danleen James Hongoro, Andre Pascal Kengne, Nasheeta Peer, Kim Nguyen, Kirsty Bobrow, Olufunke A. Alaba

**Affiliations:** 1 Health Economics Unit, School of Public Health, Faculty of Health Sciences, University of Cape Town, Anzio Road, Observatory, Cape Town 7925, South Africa; olufunke.alaba@uct.ac.za; 2 Non-Communicable Diseases Research Unit, South African Medical Research Council, Cape Town 7925, South Africa; andre.kengne@mrc.ac.za (A.P.K.); nasheeta.peer@mrc.ac.za (N.P.); kim.nguyen@mrc.ac.za (K.N.); 3 Department of Medicine, Faculty of Health Sciences, University of Cape Town, Cape Town 7925, South Africa; klbobrow@gmail.com; 4 African Population and Health Research Center, Kitisuru, Manga Close, Kirawa Road, Nairobi 00100, Kenya

## Missing Funding and Conflict of Interest Statement

In the original publication [1], the Funding statement and Conflict of Interest declaration did not fully reflect the required acknowledgement of the funder and funding administration details. The corrected Funding statement and Conflict of Interest declaration appear below. The authors state that the scientific conclusions are unaffected. This correction was approved by the Academic Editor. The original publication has also been updated.

**Funding:** This project was funded by GSK under the EDCTP2 programme (Grant Number: TMA2017GSF-1962), supported by the European Union, and administered through the South African Medical Research Council (SAMRC).
**Conflicts of Interest:** The authors declare that financial support was received for the research related to this article. This project was funded by GSK under the EDCTP2 programme (TMA2017GSF-1962), supported by the European Union, and administered through the South African Medical Research Council (SAMRC). The funders had no role in the design of the study; in the collection, analyses, or interpretation of data; in the writing of the manuscript; or in the decision to publish the results.

## References

[1] Hongoro D.J., Kengne A.P., Peer N., Nguyen K., Bobrow K., Alaba O.A. (2025). Economic Burden of Human Immunodeficiency Virus and Hypertension Care Among MOPHADHIV Trial Participants: Patient Costs and Determinants of Out-of-Pocket Expenditure in South Africa. Int. J. Environ. Res. Public Health.

