# Supplementary material for: Economic Burden of Human Immunodeficiency Virus and Hypertension Care Among MOPHADHIV Trial Participants: Patient Costs and Determinants of Out-of-Pocket Expenditure in South Africa
Source: Int J Environ Res Public Health. 2025 Sep 25;22(10):1488. doi: 10.3390/ijerph22101488 (PMC12564295; doi:10.3390/ijerph22101488)
Supplement: Supplementary file 1 [file ijerph-22-01488-s001.zip › File S1.pdf]

File SI: PATIENT COST SURVEY

Introduction to the patient:

My name is ___________________________________. On behalf of SAMRC study group we are interested in the costs that people face when they are seeking health care. Hypertension being a chronic disease that is known to be the most common reason why people visit health facilities in South Africa, this questionnaire seeks to collect information on the costs that are incurred by people with hypertension attending the HIV treatment centres. This information will be used in other studies that aim to improve hypertension care in the country.

A1.1	Patient questionnaire number			

A1.2	Name of Interviewer	
A1.3	Date of interview (dd/mm/yyyy)	__ __/__ __/__ __ __ __
A1.4	Interview start time	__ __ : __ __

A1.5	Do you want to participate to the survey	1 Yes GO to next section<br>2 No
A1.6	Reason for not participating	1. Language not good enough<br>2. Time constraint<br>3. Not comfortable<br>4. Unspecified

SECTION A: PATIENT INFORMATION

A2.1	Sex	1 Male<br>2 Female
A2.2	What is your date of birth?	_ __/__ __/__ __ __ __ (day/month/year)
A2.3	Race	1. Black African<br>2. White<br>3. Indian or Asian<br>4. Coloured<br>5. Other (specify)________________________
A2.4	Marital_Status	1 Married<br>2 Living with partner<br>3 Single/never married<br>4 Widow/widower<br>5 Separated<br>6 Divorced<br>88 Other, specify<br>___________________
A2.5	Date of First Diagnosis	__ __/__ __/__ __ __ __
A2.6	Date of first treatment	__ __/__ __/__ __ __ __
A2.7	How was the patient diagnosed?	1 Routine screening in clinic<br>2 Routine screening in community (e.g. mobile unit, health fair)<br>3 Having symptoms (e.g., headache)<br>4 Diagnosed while in clinic or hospital for some other problem<br>Other (explain): ____________________
A2.8	Other Comorbidities	1 Diabetes<br>2 Epilepsy<br>3 Asthma<br>4 Hyperlipidaemia<br>5 Rheumatoid Arthritis<br>6 Ulcerative Colitis<br>7 Hypothyroidism<br>8 Chronic obstructive pulmonary disorder<br>9 Schizophrenia<br>10 Chronic renal disease<br>11 Glaucoma<br>12 Coronary artery disease<br>13 None<br>88 Other? (specify)

Note down the current medication regime of the patient

	A2.9.1 Name	A1.9.2 Dosage/strength	A1.9.3 Frequency
a			
b			
c			
d			
e			
f			
g			

SECTION B: HEALTH SEEKING BEHAVIOUR

No	Question	Code
B1	Are you visiting this facility for the first time for hypertension?	1. Yes<br>2. No -> GO TO B3
B2	Where did you seek care previously for hypertension?	1 Hospital<br>2 Clinic<br>3 Traditional Healer<br>4 Faith Healer<br>5 Pharmacy<br>6 Health Shop<br>88 Other (Specify) _________________
B3	Why did you choose this health facility **TODAY** for your hypertension care?	1 Location close to home<br>2 Trust in providers/ high quality of care<br>3 Availability of drugs<br>4 Availability of female provider<br>5 Recommendation or referral<br>6 I was asked to come here<br>88 Other, specify: _________<br>98 Do not know
B4	What type of hypertension care are you receiving **TODAY**?	1 Pharmacy visit<br>2 Physician visit<br>3 Adherence club<br>4 All<br>88 Other____________________
B5	How many times did you visit this facility last month as a result of your hypertension?	____________________________________
B6	Did you ever have to stay long for observation as a result of Hypertension?	1 Yes<br>2 No -> GO TO SECTION C
B7	How long did you stay?	___________hours

SECTION C: TRAVEL COSTS AND WAITING TIME

I will now ask you some questions related to your visit **TODAY**. These questions are related to your mode of travel, how long it took you to come here, and how it affected your usual activities.

No	Question	Code
C1	How did you travel to this health facility today?	1 Public transport<br>2 Car (private transport)<br>3 Walking<br>4 Cycling<br>88 Other (specify)<br>98 Don't know
C2	How many minutes did you take to get to this facility **TODAY** from your house (one-way)?	__________minutes
C3	When you came to the facility **TODAY**, did anyone accompany you here such as family or a friend?	1 Yes -> GO TO C7<br>2 No

Question No	C4. What is your relationship with that person?	C5. Did they miss school/work because of this clinic visit?	C6. How much does the person accompanying you earn per day?	C7. How many hours of school did they miss?
Person 1	1 Relative<br>2 Friend<br>3 Child<br>4 Care giver<br>5 Other (specify)	1 Yes<br>2 No	1 Nothing<br>2 R___________	_________hrs
Person 2	1 Relative<br>2 Friend<br>3 Child<br>4 Care giver<br>5 Other (specify)	1 Yes<br>2 No	1 Nothing<br>2 R___________	_________hrs
Person 3	1 Relative<br>2 Friend<br>3 Child<br>4 Care giver<br>5 Other (specify)	1 Yes<br>2 No	1 Nothing<br>2 R___________	_________hrs

C8	At what time did you arrive at this facility **TODAY**?	__ __/__ __ Hrs	
C9	How much did you pay to come to this health facility **TODAY** (one-way)?"	1. Nothing<br>2. R___________	notes
C10	If you did not have to come to this facility, what would you be doing **TODAY**?	1 Working<br>2 Relaxing at Home<br>3 Working at Home<br>4 Looking after Children<br>5 Other? (specify) ____________	
C11	What are you doing after this clinic visit?	1 Going Home *Skip to Section **D***<br>2 Going to work<br>88 Other (specify)? _____________<br>98 Don't Know	
C12	Will you get your normal day's wage for today?	1. Yes<br>2. No -> GO TO C14	
C13	Will you able to work a full day?	1 Yes -> If YES skip to section D<br>2 No	
C14	How many hours will you able to work **TODAY**?	______________hours	

SECTION D: TREATMENT COSTS

I will now ask you some questions about the costs you incurred. These will include treatment costs, travel costs, food costs and costs to the people accompanying you, if any.

About how much did you spend on each of the following items during **TODAY's** visit *(for all that don't apply mark N/A)*

D1	Administration fees	R________________________
D2	Consultation fees	R________________________
D3	Laboratory tests	R________________________
D4	Medication	R________________________
D5	Under the table fees	R________________________

SECTION E: FOOD AND GUARDIAN COSTS

Nº	Question	Code
E1	When you came did you bring something to eat?	1. Yes<br>2. No
E2	If No, did you buy anything to eat?	1 Yes<br>2 No -> GO TO *E4*
E3	How much did you pay in total for the food?	R________________
E4	Did you get someone to look after your children or the house while you visited the clinic? (i.e. guardian or caregiver)	1. Yes<br>2. No -> GO TO *Section F*
E5	How much are you paying them?	R___________________

SECTION F: MEDICATION COMPLIANCE AND ADHERENCE

Nº	Question	Code	
F1	During the past month, did you on any occasion not take your blood pressure medication?	1. Yes -> GO TO F3<br>2. No	
F2	If **NO**, what made you remember to take your medication on time?	1. Caregiver<br>2. Reminders (e.g. alarm)<br>3. Adherence clubs<br>4. Family<br>5. Headache	6. Phone app<br>7. Feeling sick<br>8. I just knew<br>88 Other (please specify)<br>98 Don't know
F3	In the past month, did you at any moment feel the need to stop taking hypertension medication?	1. Yes<br>2. No -> GO TO F5	
F4	Why did you feel like not taking the tablets?	1. I was feeling fine<br>2. Tired of taking medication.<br>3. Too much medication<br>4. Side Effects	5. Consulting with other health care practices (e.g. traditional healers)<br>88 Other (specify)<br>______________________<br>98 Don't know
F5	Thinking back last month, did you take anything else other than your normal hypertension tablets to	1. Yes<br>2. No<br>98 Don't know	

	control your hypertension? E.g. vitamins	
F6	How long will the supply of medicine that you received **TODAY** last?	1. One Month<br>2. Two Months<br>3. Three Months
F7	Have you ever used your phone to search for health information?	1. Yes<br>2. No
F8	Are there any other costs that you incurred as a result of **TODAY'S** visit that I haven't asked?	1. Yes<br>2. No<br>98 Don't know

Please list the costs and their respective amounts below.

	F9 **Cost**	F10 **Amount** (ZAR)
a		
b		
c		

SECTION G: SOCIO-ECONOMIC INFORMATION

G1	What is your employment status?	1. Employed full-time<br>2. Self-employed (formal sector)<br>3. Part-time/contract/temporary<br>4. Casual<br>5. Self-employed (informal sector)<br>6. Unemployed<br>7. Housewife<br>8. Pensioner<br>9. Student/learner/child<br>10. Disabled and unable to work<br>98. Don't know<br>88 Other (specify) ______________
G2	Is the reason for your unemployment your chronic illness (hypertension)?	1 Yes<br>2 No
G3	If yes, when was the last time you were working? (month/year)	
G4	Are you receiving any income?	1 Yes<br>2 No
G5	What is your source of income?	1 Working at a full time job<br>2 Working at a part time job<br>3 Social Grant<br>4 I receive money from other people
G6	What was your total average monthly income **PRIOR** to your diagnosis with hypertension?	R____________________
G7	What is your income **NOW**?	R__________/Day<br><br>R__________/Week

		R__________/Month
G7	Have you ever stopped working/going to school/doing housework due to your hypertension?	1 Yes<br>2 No -> GO TO G9
G8	If yes, for how long?	1 Less than 1 month<br>2 One month<br>3 2-3 months<br>4 4-5 months<br>5 More than 6 months
G9	Do you have children?	1 Yes<br>2 No -> GO TO G11
G10	How many children do you have?	1 One<br>2 Two<br>3 Three<br>4 Four<br>5 More than five
G11	What is your relation to the household head?	1. Me<br>2. Husband/wife/partner<br>3. Son/daughter<br>4. Adopted son/daughter<br>5. Stepchild<br>6. Brother/sister<br>7. Parent (mother/father)<br>8. Parent-in-law<br>9. Grand/great grandchild<br>10. Son/daughter-in-law<br>11. Brother/sister-in-law<br>12. Grandmother/father<br>13. Other relative<br>Non-related person
G12	What is your highest level of education?	1 No formal education<br>2 Primary education<br>3 Secondary education<br>4 Tertiary education<br>88 Other (specify) ___________
G13	Are you covered by a Medical Aid or Medical Benefit Scheme or any scheme that helps you pay for health-care services or medicines?	1 Yes<br>2 No

SECTION H: COPING COSTS

I will now ask you some questions about how you cover the cost of your care.

Nº	Question	Code
H1	Did you borrow any money when you came for this clinic visit?	1. Yes<br>2. No -> GO TO H4
H2	How much did you borrow?	R _________________
H3	Does the money have interest?	1. Yes<br>2. No -> GO TO H5

H4	How much interest do you have to pay?	R______________
H5	Did you take up any extra work to cover the costs for **TODAY'S** visit?	1. Yes<br>2. No
H6	Was there anything that you sold to cover the costs for **TODAY'S** clinic visit i.e. jewellery, radio, television or furniture?	1. Yes<br>2. No -> GO TO H9
H7	How much did you get from the sale?	R____________________
H8	For **TODAY'S** clinic visit, did you withdraw any money from your savings account?	1. Yes<br>2. No -> GO TO H10
H9	How much did you withdraw?	R_______________________
H10	Did you have to modify your diet due to Hypertension?	1 Yes<br>2 No
H11	Is this diet modification costing you any money?	1 Yes<br>2 No
H12	How much is this diet modification costing you per month?	R____________________

Thank You for participating in the survey!
