# Supplementary material for: Economic Burden of Human Immunodeficiency Virus and Hypertension Care Among MOPHADHIV Trial Participants: Patient Costs and Determinants of Out-of-Pocket Expenditure in South Africa
Source: Int J Environ Res Public Health. 2025 Sep 25;22(10):1488. doi: 10.3390/ijerph22101488 (PMC12564295; doi:10.3390/ijerph22101488)
Supplement: Supplementary file 1 [file ijerph-22-01488-s001.zip › File S2.pdf]

File S2. Econometric Specifications

GLM Model

We estimated the determinants of total patient-incurred costs using GLM with a gamma distribution and log link, which are well suited for modeling right-skewed, non-negative outcomes. The GLM specification is expressed as:

$$E(Yi \mid Xi) = exp(\beta 0 + \beta 1X1i + \beta 2X2i + \cdots + \beta kXki) \qquad (8)$$

where Yi represents the monthly total cost incurred by patient *i,* X*i* is a vector of explanatory variables, and β are parameters estimated using quasi-likelihood methods. To aid interpretation, we computed average marginal effects (AMEs), which quantify the expected change in mean costs associated with changes in each covariate.

OLS Sensitivity Models

As a robustness check, we also estimated ordinary least squares (OLS) models on log-transformed costs:

$$ln(Yi + 1) = \alpha 0 + \alpha 1X1i + \alpha 2X2i + \cdots + \alpha kXki + \epsilon i \qquad (9)$$

where coefficients α are interpreted as semi-elasticities, representing the percentage change in costs associated with a one-unit change in the covariate. The OLS results are presented only as sensitivity analyses and are clearly distinguished from the primary GLM results. Model fit was assessed using Akaike Information Criterion (AIC), Bayesian Information Criterion (BIC), scaled deviance, and residual diagnostics.

Sensitivity Analysis

To assess robustness, we estimated OLS regressions on log-transformed cost variables:

$$\ln(C_i + 1) = X_I\beta + \varepsilon_I \qquad (10)$$

Where ε_I is the error term assumed to be normal. Both OLS models included the same predictors as the GLM specification and used heteroskedasticity-robust standard errors.

Cost Burden and Catastrophic Expenditure

We evaluated the cost burden as a share of income:

$$B_i = \left(\frac{C_{total\_actual}}{I_i}\right) * 100 \qquad (11)$$

Where B_i is the percent burden for individual i and Ii is their monthly income. Catastrophic expenditure was defined for each patient as:

$$Cat_i^{(t)} = \begin{cases} 1\ if\ B_i > t \\ 0\ otherwise \end{cases} \qquad (12)$$

for thresholds t = {10%, 20%, 25%, 30%, 40%}. Proportions of individuals exceeding each threshold were tabulated
